# Please stay seated – the specialist AI will see you shortly

**DOI:** 10.1055/a-2655-6281

**Published:** 2025-08-06

**Authors:** Keith Siau

**Affiliations:** 1 8028 Gastroenterology, Royal Cornwall Hospitals NHS Trust, Truro, United Kingdom of Great Britain and Northern Ireland; 2 150183 Immunology and Immunotherapy, University of Birmingham College of Medical and Dental Sciences, Birmingham, United Kingdom of Great Britain and Northern Ireland



The adage “garbage in, garbage out” is an important reminder in the era of artificial intelligence (AI), particularly with increasing adoption in healthcare. General-purpose large language models (LLMs) such as ChatGPT, Bard, and Claude have gained popularity among clinicians due to their ability to convert prompts into meaningful, human-like responses within seconds. However, their reliance on training from a sea of unvetted internet-based data spanning journals, news articles, blogs, and social media platforms means that their outputs should be approached with caution for clinical matters.

Developing a specialized LLM requires curation of high-quality, clinically relevant data. The evidence base is polluted with potential garbage in the form of flawed methodology, invalid abstracts, and even conflicting guidelines, and without expert filtering, this noise of information risks being encoded into AI outputs. Specialized development allows for expert-guided curation, emphasizing high-quality evidence from reputable sources such as guidelines and high-quality studies, including paywalled content inaccessible to general LLMs.

In this issue, Simsek and colleagues present data on their gastroenterology-specific LLM termed GastroGPT, compiled using 1.2 million tokens from gastroenterology journals, guidelines, and textbooks. In a blinded comparison across 10 simulated gastroenterological scenarios, GastroGPT significantly outperformed general LLMs of ChatGPT 4, Bard, and Claude, with an overall expert rating of 8.1 out of 10 vs 5.2 to 7.0 for general LLMs. GastroGPT was particularly superior for tasks demanding structured clinical reasoning, such as selecting diagnostic investigations or applying guidelines to management. Moreover, its lower variance in scores (34.95 for GastroGPT vs 97.4 to 260.35 for general LLMs) suggests superior consistency with model outputs, which can increase user confidence in AI-assisted decision-making.

The case for specialized AI has been demonstrated in various sectors. In finance, BloombergGPT, a 50 billion-parameter model trained exclusively using financial data, achieves unrivalled results in monetary tasks. Within biomedicine, BioGPT has been pretrained on 15 million PubMed abstracts to better understand specialized vocabulary. Across healthcare, a myriad of LLMs exist but focus mainly on specialist roles such as image interpretation. Google’s Med-PaLM 1 , a 540 billion-parameter LLM, was the first system to surpass the 60% passmark for the United States Medical Licensing Examination but remained inferior to clinicians. The examples show that domain-specific training legitimately produces better outcomes than asking general-purpose models to handle specialized tasks. This validates the shift from use of general LLMs toward precision-engineered, specialty-focused models exemplified by GastroGPT.

Limitations of this study include its small numbers, potential conflict of interest with co-authors related to development of GastroGPT, and lack of comparisons with humans or, indeed, a medical LLM such as Med-PaLM. There is limited transparency regarding specific pretraining data sources (including paywalled assets), prompt engineering, and data processing. It is possible that the model could have been developed with the quality domains or “mark scheme” in mind, and it is unclear whether performance differences reflect genuine model capabilities versus prompt optimization favoring GastroGPT. As such, larger studies with real-world clinical validation remain necessary.

Just as medical training differentiates between generalists and specialists, a similar distinction is emerging within the field of AI. GastroGPT is an exciting proof of concept for the value of domain-specific, task-oriented AI in clinical medicine. As AI continues to evolve, the maxim “garbage in, garbage out” should be replaced with “quality in, quality out”, aiming to develop specialized AI systems, akin to consultant (specialist) level, aligned with the credibility and rigor of the role in medicine.
